# High Aspect Ratio Structuring of Glass with Ultrafast Bessel Beams

**DOI:** 10.3390/ma14226749

**Published:** 2021-11-09

**Authors:** Christian Vetter, Remo Giust, Luca Furfaro, Cyril Billet, Luc Froehly, Francois Courvoisier

**Affiliations:** FEMTO-ST Institute, University Bourgogne Franche-Comté, CNRS, 15B Avenue des Montboucons, CEDEX, 25030 Besançon, France; christian.vetter@iof.fraunhofer.de (C.V.); remo.giust@univ-fcomte.fr (R.G.); luca.furfaro@univ-fcomte.fr (L.F.); cyril.billet@univ-fcomte.fr (C.B.); luc.froehly@univ-fcomte.fr (L.F.)

**Keywords:** glass, ultrafast laser processing, Bessel beam

## Abstract

Controlling the formation of high aspect ratio void channels inside glass is important for applications like the high-speed dicing of glass. Here, we investigate void formation using ultrafast Bessel beams in the single shot illumination regime. We characterize the morphology of the damages as a function of pulse energy, pulse duration, and position of the beam inside fused silica, Corning Eagle XG, and Corning Gorilla glass. While a large set of parameters allow for void formation inside fused silica, the operating window is much more restricted for Eagle XG and Gorilla glass. The transient formation of a molten layer around voids enables us interpreting the evolution of the morphology with pulse energy and duration.

## 1. Introduction

Cutting and drilling glass is an important technological problem, particularly when the width over which the material has to be modified is much smaller than the length of the modification. This is high aspect ratio structuring. In this context, ultrafast laser processing is very attractive because the infrared wavelength and the very high intensity of the pulses enable energy deposition in three dimensions, including inside the bulk of glass [1]. The laser-induced micro/nano-structures can be index modifications, nanogratings or even voids [2]. These modifications can be combined with chemical etching, which led to a number of advances in the field of photonics, microfluidics chips, mechanics [3,4].

Because point-by-point scanning is relatively slow, a number of techniques were developed to improve the overall speed for the micro-structuring. In addition, elongated voids cannot be formed by scanning, since a nano-void produced by a first pulse would be erased by the microexplosion produced by the second pulse next to it [5]. Bessel beams were revealed to be excellent candidates to control the formation of high aspect ratio elongated structures inside transparent materials [6,7]. Bessel beams are formed by a conical superposition of plane waves, where the central hotspot maintains its diameter over the whole length of the interference region [8,9]. Importantly, when the focusing angle of the Bessel beam is sufficiently high, the conical flow of energy crosses the high-intensity region only in the central hotspot. This drastically reduces the influence of the Kerr effect, so that longitudinally-invariant structures can be processed within dielectrics [10,11,12].

Laser structuring with high aspect ratio is particularly useful in the context of stealth dicing, a concept in which a series of pulses under high-speed translation of the sample is used to create an entire modification plane [13]. A mechanical, chemical, or thermal stress is then used to cleave the glass along the modified plane. The main benefit of this approach is that cutting is possible at very high speed (typically 10 cm to 1 m per second). Filamentation, i.e., ultrafast pulse self-channeling in glass, was first used for stealth dicing purposes [14,15]. Bessel beams offer in addition the possibility to control very easily the onset of the material damage by simple geometrical considerations since nonlinear effects are reduced. They were used to cut glass and sapphire [16,17,18,19,20]. Since beam length and focusing angle can be independently adjusted, it is also possible to produce very long Bessel beams with the same angle that allows for cutting thick glass. This however requires high pulse energies since the energy is spread over the entire focal line of the beam [21]. With millijoule energy, Bessel beams were used to cleave glass up to 1 cm thick [22,23]. Recently, breaking the symmetry of the Bessel beam was used to improve the precision and ability of guiding fractures [24,25,26,27]. Controlling the formation of voids with ultrafast laser pulses is key to improve the stealth dicing of glass.

Here, we show that, depending on the material, voids do not form for the same parameters. We compare three glass types: fused silica, Corning${}^{®}$ Eagle XG${}^{®}$, and Corning${}^{®}$ Gorilla${}^{®}$ glass. We investigate the regimes in which various structures are produced using single shot Bessel beams depending on pulse energy, pulse duration, and position in the sample. We observed that these three parameters control the formation of various types of structures inside glass. Future development of applications such as laser cutting should therefore operate in the right parameter window.

## 2. Materials and Methods

**Figure 1:**
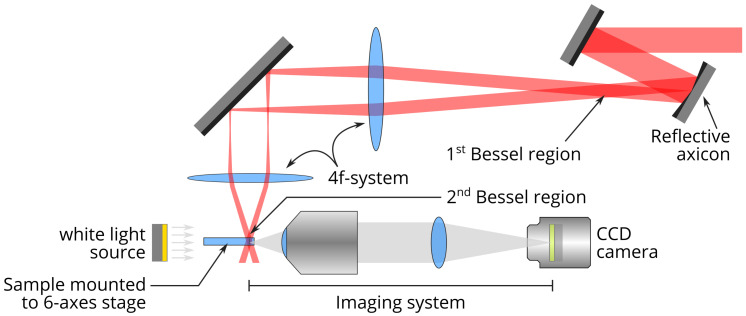
Experimental setup. Ultrafast laser beam is shaped by a reflective axicon (Cailabs). It is then demagnified by a telescopic arrangement in a 2f-2f configuration. Laser-written structures are observed using an incoherent white light source.

**Figure 2:**
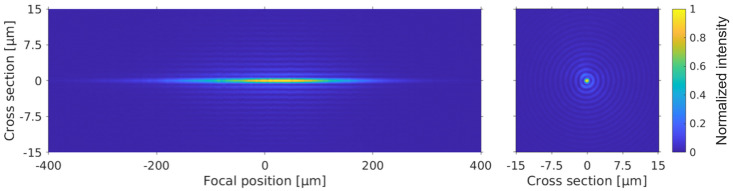
Characterization of experimental Bessel beam. (**left**) Longitudinal cross-cut of fluence distribution as a function of relative propagation distance in air. Please note aspect ratio of axes. (**right**) Cross-section of fluence distribution at center of beam.

Our experimental setup (see Figure 1) is composed of a chirped pulse amplified (CPA) Ti-Sapphire laser source, at 800 nm central wavelength and of a reflective axicon (Cailabs) associated to a telescope arrangement to magnify the cone angle [28]. The laser pulses can be stretched from 100 fs to 3 ps using the grating compressor of the amplifier. The experimental characterization of the beam is shown in Figure 2. The Bessel beam has a cone angle of 19.5${}^{{}^{\circ}}$ in air, which corresponds to a central spot diameter of 0.9 μm Full Width at Half Maximum (FWHM). The Bessel beam length is ∼220 μm FWHM. One can notice the high degree of circular symmetry of the Bessel beam. All samples were 700 μm thick.

**Figure 3:**
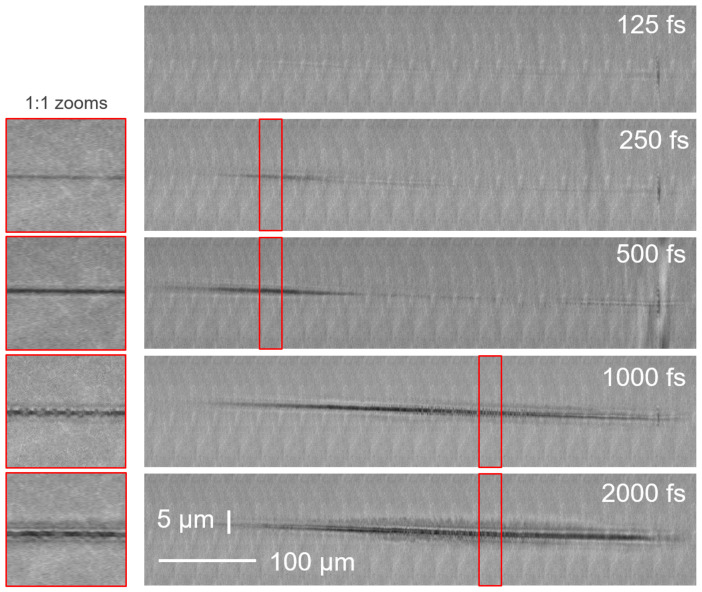
Microscope images of damages produced in fused silica glass for several pulse durations, for same beam position inside sample and for a constant single shot energy of 52 μJ. Please note image aspect ratios (left insets show 1:1 zooms of panels to their right). Beam propagates from left to right.

After processing, the laser-modified regions were characterized by scanning the sample with white light, brightfield illumination, in an optical microscope arrangement with a numerical aperture of 0.8 and aberration-correction. Stitching the microscope images provided high resolution images of the whole high aspect ratio structure. Figure 3 shows the result of single shot illumination in fused silica at a pulse energy of 52 μJ, for increasing pulse durations. A periodic background pattern can be observed and simply arises from the illumination inhomogeneity associated to the stitching operation.

## 3. Results

### 3.1. Microstructure Types

**Figure 4:**
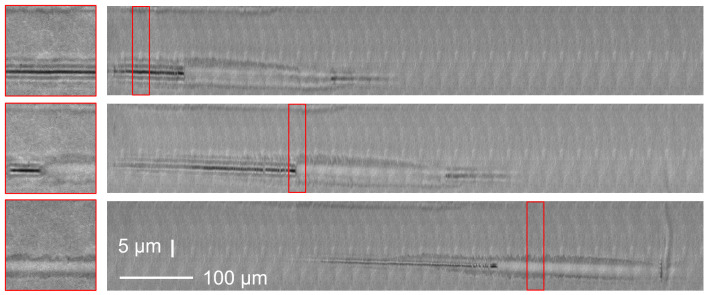
Brightfield microscopy images of damages produced in Gorilla glass with 3 ps pulse duration at a constant energy of 52 μJ as in Figure 4. Position of beam in sample was varied from top to bottom. Please note image aspect ratios (left insets show 1:1 zooms of panels to their right).

In Figure 3, at a pulse duration of 125 fs, only a line of faint index change is observable. In the cases of 250 and 500 fs, high- aspect ratio voids are observed, they are characterized by black marks. We readily see that the void structure is shorter than the Bessel beam length. The void is present only in the moderate intensity region while in the highest intensity region, the void structure has almost vanished. From 1 ps, the black mark is split around its center into small dots: these are void bubbles (see scanning electron microscopy image in Figure 4 of reference [29]). This process is called bubbling hereafter. Faint bubbling appears here in the central region of the Bessel beam and is slightly stronger for the case of 2 ps.

Importantly, the morphology of the material transformation can vary along the beam. An example is shown in Figure 4 where we report the results for the interaction of a 3 ps Bessel beam in Gorilla glass for different focusing positions inside the sample. In this example, the material modification undergoes minor variations with the position. We can observe three sections with different morphologies from left to right: (i) a void with faint bubbling in the relatively low intensity region of the Bessel beam (onset), (ii) a wide index modification in the highest intensity segment of the Bessel beam, and (iii) a void. The central panel allows us to understand the ordering of the material modification type with increasing intensity: index modification on a small diameter (central lobe), void formation, structure with more or less randomly distributed bubbles, wide index index modification on a diameter much larger than the beam central lobe. The wide modification is developed longitudinally step-wise, suggesting a threshold-based mechanism. It erased all structures like void or bubbles. Note that the bubbles appear elliptical because we compressed the horizontal axis scale by a factor 5.

**Figure 5:**
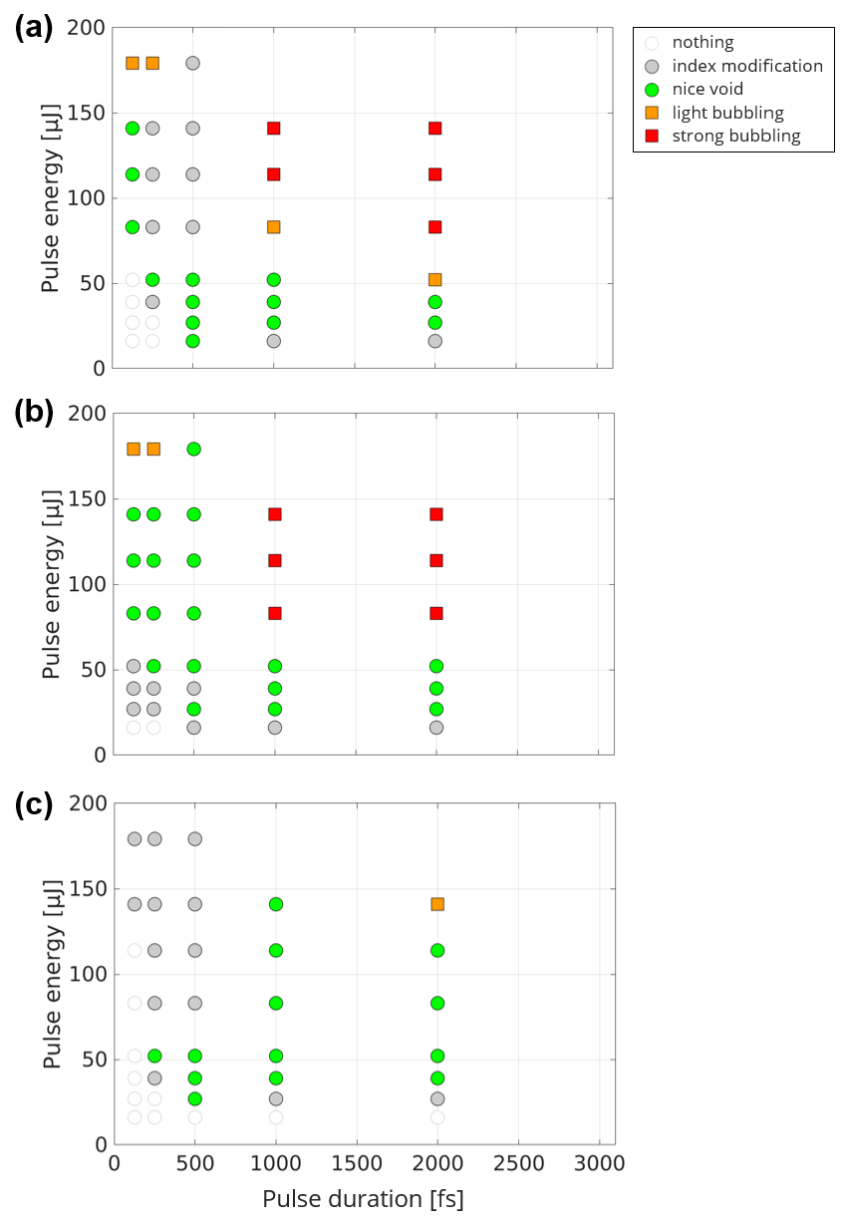
Morphology of single shot-induced damage in fused silica as a function of pulse energy and pulse duration. This is performed for a Bessel beam position: (**a**) at center of sample, (**b**) crossing the entrance surface, (**c**) crossing exit surface.

We further explored, in a parametric study, the evolution of the laser-induced structures as a function of pulse energy, pulse duration, and beam position. We summarized our results in Figure 5 for three positions of the Bessel beam inside the fused silica sample. In Figure 5a, the beam is positioned at the center of the sample such that none of the entrance and exit surfaces are damaged. We classified the modifications in 5 groups, ranging from no damage to strong bubbling. We note that we did not make a separate class for the wide index modification, such as the one shown in Figure 4, because those modifications are conventionally useless for applications. Also, they often occur alongside voids, which we are more interested in. For the drilling and cutting of glass applications, the narrow index modifications and nice voids, shown respectively as gray and green markers, are the most interesting features. When bubbling occurred on a substantial section of the beam, the structure was classified as bubbling.

In Figure 5a, we see that the relatively large range of parameters allow for inducing nice voids in fused silica, similarly as in reference [30]. Higher energies and longer pulse durations tend to create bubbling. When the pulse duration is increased, the energy required to open a void is reduced, but bubbling also arises earlier. The energy range over which nice voids can be opened is reduced at longer pulse durations. In Figure 5b, the beam crosses the entrance side. In this case, the shortest pulse durations provide conditions where voids can be opened on a larger range of parameters. The presence of the interface increases the ability of void opening after energy deposition, as was already noticed in a former reference [6]. In Figure 5c, the beam crosses the exit interface. In this case, the short pulse durations induce voids with very small diameters. A much larger range of energies and pulse durations enable void formation. In almost all the cases tested here, nice voids could be produced. We hypothesize that our results can be understood by the following: the presence of the interface allows the phenomenon of ablation cooling [31] where a fraction of the energy deposited inside the material can be evacuated via the open channel at the surface. This allows for a fast quenching of the material relaxation which increases the capability of “freezing” a void before the compressed melt part surrounding the void can close it. In the case where the beam crosses the entrance surface (Figure 5b), the dynamics of plasma formation is slightly different from the two other cases because a dense plasma can form on the surface which can then shield light preventing void formation in the very first micrometres from the surface. This could prevent the effect of the ablation cooling described above.

### 3.2. Other Glasses

**Figure 6:**
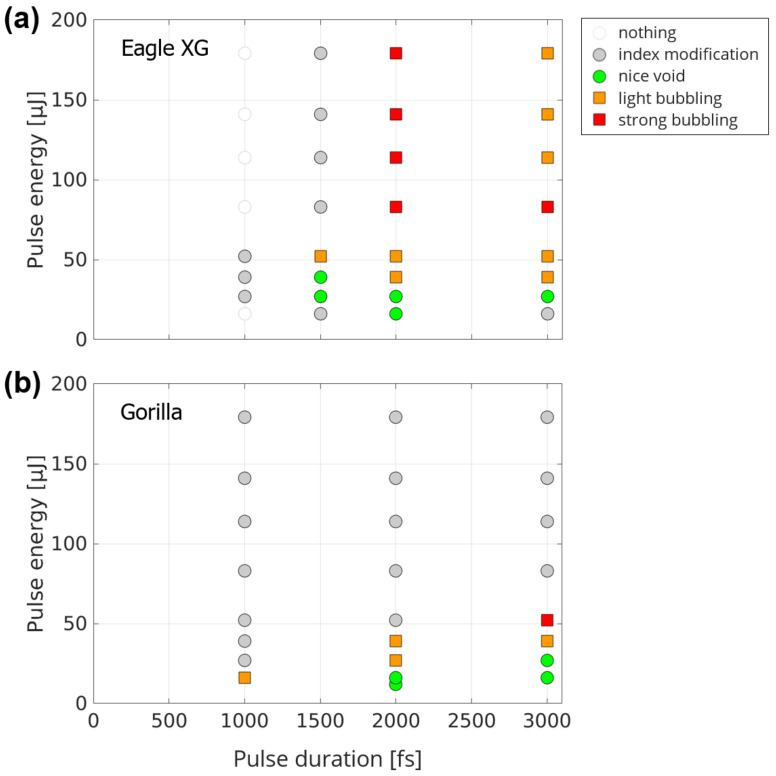
Morphology of single shot-induced damage in (**a**) Eagle XG and (**b**) Gorilla glass as a function of pulse energy and pulse duration. Beam is centered inside sample.

In Figure 6, we show the results for Eagle XG glass (Figure 6a) and Gorilla glass (Figure 6b) when the beam is enclosed within the sample, as in Figure 5a. For these two materials, the situation highly differs from the case of fused silica. Overall, it is much more difficult to create voids, particularly for short pulses, where no modification is apparent below a threshold pulse duration of 1 ps.

The operating window for void formation is restricted to a small energy range before bubbling sets in. This window is even smaller in the case of Gorilla glass. For most of the pulse durations in Gorilla glass, above the energy for the bubbling regime, only faint structures are found, whereas this phenomenon occurs in Eagle XG only for 1.5 ps and never in fused silica. It could be attributed to a slower relaxation in Gorilla glass than in that of the other media.

We attribute the differences between the different glasses in terms of void opening to the difference in softening point. Indeed, it exceeds 1500 ${}^{{}^{\circ}}$C for fused silica, while it is only of 971 ${}^{{}^{\circ}}$C for Eagle XG, and 853 ${}^{{}^{\circ}}$C for Gorilla glass. Therefore, assuming a similar profile of laser-deposited energy, the molten region is wider in the case of Eagle XG and Gorilla glass than in the case of fused silica. This would lead to a faster quenching of the relaxation in fused silica, with stronger gradients, in contrast with the other two glasses.

In Eagle XG and Gorilla glass, beam positioning close to the entrance and exit surfaces yield to similar tendencies as in the case of fused silica. While no major differences could be observed with Figure 6 when the beam crosses the entrance surface, we observed a much larger operating window for void opening when the beam crosses the exit surface for both glass types.

### 3.3. Large Heat-Affected Zone for Picosecond Pulses

**Figure 7:**
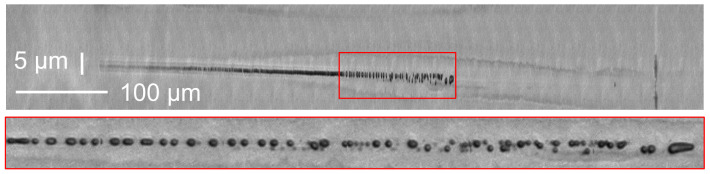
Strong bubbling in Eagle XG glass. Pulse duration is 3 ps, pulse energy 141 μJ. Please note image aspect ratio. Bottom inset shows a 1:1 zoom of marked area in panel above.

Figure 7 shows the result of a 3 ps laser shot in Eagle XG. One can notice that the heat affected zone is very wide, typically above 8 μm in diameter. Importantly, in this regime, the bubbles are not aligned: it seems that they have randomly moved from the optical axis during the relaxation stage. This well supports the interpretation of a large molten layer around the void at large pulse energies and duration.

## 4. Conclusions

We observed that void formation in transparent glasses strongly depends on energy and pulse duration. In fused silica, voids can be formed for a relatively large set of parameters. By increasing pulse energy and pulse duration, the high aspect ratio void channel splits into bubbles with a diameter of approximately 1 μm. This structure can even vanish for higher energies. In Eagle XG and Gorilla glass, only very long pulse durations could yield void formation, but for a very limited energy range. For a pulse duration of 2 ps and 16 μJ pulse energy, a void can be formed in all three materials, with a length of 150 ± 10 μm in Eagle XG, of 170 ± 10 μm in fused silica and the highest length is for Gorilla glass with 230 ± 10 μm. For all materials, we remark that void formation is possible over a larger range of energies when the beam crosses the exit surface.

We interpret the evolution of the morphology of the damages induced by ultrashort Bessel beams in these glasses in the following way. For sufficiently high energies, a void can form inside glass, either after a micro-explosion [32,33] or by cavitation inside a molten zone [34]. We infer that this void can be transiently surrounded by a molten layer, whose thickness depends on the glass type and pulse duration. The longest pulse durations and the highest energies tend to increase the thickness of the molten layer. A low softening point, such as in the case of Eagle XG and Gorilla also increase the molten layer. Thicker molten layers increase the occurrence of bubbling or are even closing the void. We understand the formation of bubbles as a redistribution of the material during the cooling stage by splitting or even fully closing the void transiently formed. We interpret the role of the exit surface as a cooling agent. The evacuation of a fraction of the hot material via the void channel produced at the center of the Bessel beam, allows for quenching the relaxation and maintains the void structure even for parameters where the void would be partially or totally reclosed by the molten material around it. Recent results on double pulse illumination let us anticipate that double pulse could partially reduce the amount of the molten layer, and increase the temperature contrast between the optical axis and the surrounding lobes [35].

These results shed light on the formation of high aspect ratio void channels inside different glasses. We believe they will impact on future technologies for ultrafast laser material processing of glass.

## Data Availability

The data presented in this study are available upon reasonable request from the corresponding author.
